# The aetiology of community associated pneumonia in children in Nanjing, China and aetiological patterns associated with age and season

**DOI:** 10.1186/s12889-015-1422-1

**Published:** 2015-02-10

**Authors:** Keping Chen, Runqing Jia, Li Li, Chuankun Yang, Yan Shi

**Affiliations:** Zhongda Hospital, Southeast University, 87 Dingjiaqiao, Nanjing, 210009 Jiangsu China; College of Life Sciences and Bioengineering, Beijing University of Technology, 100 Ping Le Yuan, Beijing, 100022 China

**Keywords:** Community-acquired pneumonia, Aetiology, *M. pneumoniae*, Pneumoslide IgM

## Abstract

**Background:**

Viral and atypical bacterial pathogens play an important role in respiratory tract infection. Using the Pneumoslide IgM test, the presented study explored the aetiology of community-acquired pneumonia and investigated further whether there was an association between age or season and aetiological organisms.

**Methods:**

Serum samples, taken between August 2011 and August 2013, from patients with CAP were tested with the Pneumoslide IgM kit. The Pneumoslide IgM technology can simultaneously diagnose 9 viral and atypical bacterial pathogens: *Legionella pneumophila* serogroup 1 (LP1), *Mycoplasma pneumoniae* (MP), *Coxiella burnetii* (COX), *Chlamydophila pneumonia* (CP), Adenovirus (ADV), Respiratory syncytial virus (RSV), Influenza A (INFA), Influenza B (INFB), Parainfluenza 1, 2 and 3 (PIVs). The data was analyzed by using Statistical Package for the Social Sciences for Windows (SPSS, version 11.0).

**Results:**

Of a total of 1204 serum samples tested, 624 samples were positive. *M. pneumoniae* was the dominant pathogen, with INFB, PIVs, and RSV ranking second to fourth, respectively. The positive percentages of MP, INFB, PIVs and RSV were found to be associated with age, especially MP, INFB and PIVs. The positive percentages of MP, PIVs and RSV were also found to be associated with season. The positive percentage of MP in autumn was the highest. The positive percentages of LP1 in August and September, ADV in June and INFB in March were relatively higher than that in other months.

**Conclusions:**

The results show there were 4 main viral and atypical bacterial pathogens causing CAP in our study. Some pathogens were found to be associated with age and season. *M. pneumoniae* was the most predominant pathogen among these 9 pathogens. It is necessary to take preventative measures in order to prevent the spread of these pathogens in susceptible age groups during peak season.

## Background

Community-acquired pneumonia (CAP) refers to pneumonia acquired outside of a health care facility. In the United States, CAP is the number-one cause of death from infection and the sixth leading cause of death overall. Each year, it is responsible for about 4.2 million outpatient visits and more than 60,000 deaths [1,2]. It is important to understand the possible causes of CAP and which are most likely to occur so that appropriate therapies can be selected. The primary pathogens responsible for CAP broadly include typical bacterial pathogens, atypical bacterial pathogens and viruses [3]. With the development of new laboratory testing technologies in recent years, it was found that the roles of viral and atypical bacterial pathogens in respiratory tract infection were underestimated. Research shows that about 33.3% of CAP cases are caused by viral and atypical bacterial pathogens [4]. Choi, et al. studied 198 patients with pneumonia and the determination was that 35% of the patients had a bacterial infection and 36% had viral infection [5]. In 2010, pneumonia was ranked in the United States as the sixth leading cause of death in children 1 to 4 years of age [6]. It is estimated that for every 1000 infants and children in North America and Europe, 35 to 40 will be affected by CAP [7]. Viral pathogens are the most common cause of CAP in children younger than 2 years of age, accounting for 80% of cases [8].

The prevalence of each pathogen varies from country to country and could be due to differences in seasons and geographic areas. Nanjing is the provincial capital of Jiangsu, and is a metropolitan area with a population greater than 7 million people, so exploring the aetiology of pneumonia in Nanjing is significant for the health of local children because of their immature and susceptible immune defenses.

A rapid and standardized diagnostic method for the detection of pathogens in children with CAP is important. The currently validated methods to define the etiology of infection are serology, cell culture and PCR. Viral culture can also be employed for most of the respiratory viruses, but the need for specific culture medium and the lengthy diagnosis times are substantial disadvantages [4]. Recently, PCR has been reported as a rapid method with high sensitivity that may exceed that of culture, but PCR assays need specialized equipment and the reagents are expensive [9]. Much research shows that the Pneumoslide IgM test is a reasonably sensitive, highly specific, easy, rapid and cost-effective technique for detection of viral or atypical bacterial pathogens [10]. As an indirect immunofluorescense technique for IgM detection, Pneumoslide IgM can simultaneously diagnose 9 pathogens of infectious disease of the respiratory tract, including the *Legionella pneumophila* serogroup 1 (LP1), *Mycoplasma pneumoniae* (MP), *Coxiella burnetii* (COX), *Chlamydophila pneumoniae* (CP), Adenovirus (ADV), Respiratory syncytial virus (RSV), Influenza A (INFA), Influenza B (INFB), Parainfluenza 1, 2 and 3 (PIVs). Pneumoslide IgM could detect virus in 25% of patients, whereas viral culture detected only in 16.7%. Sally compared the technique with PCR, and reported the sensitivity and specificity of Pneumoslide IgM for RSV was 75% and 98.1%, respectively, whereas they were 78% and 95% for M. pneumoniae, respectively [10]. So Pneumoslide IgM had reasonable sensitivity and specificity for detection pathogens causing CAP. The presented investigation studies the aetiology of CAP treated in Zhongda Hospital and further investigates whether there was an association between age or season and the aetiological organism, which can assist in planning better therapeutic and prevention strategies to prevent the spread of the aetiological organism in susceptible age groups during peak season.

## Methods

### Subject information

The study was conducted on 1204 children patients suffering from CAP recruited from Zhongda Hospital, Southeast University, Nanjing, China. Written informed consent was obtained from all participants’ guardians. The study was approved by the Human Investigation Review Committee at Zhongda Hospital Affiliated to Southeast University. Diagnosis of pneumonia followed World Health Organization Criteria (1994). All of the child patients showed signs and symptoms of pneumonia caused by an infection that had been acquired outside the hospital.

Of the patients, 715 children were male and 489 were female. Aged 4 h after birth to 14 years old, children were divided into four groups: infants group (newborn ~1 year old), 184 cases; toddlers group (>1 ~ 3 years old), 477 cases; preschool group (>3 ~ 6 years old), 403 cases; school children group (7 ~ 14 years old), 140 cases. Table 1 shows the socio-demographic profile of the children in this study. Serums samples of patients were separated from venous blood drawn from every child and tested with Pneumoslide IgM to explore the aetiology of CAP. According to the seasons when the Pneumoslide IgM tests were performed, all the subjects were divided into four group: spring group (March, April and May), 316 cases; summer group (June, July and August), 295 cases; autumn group (September, October and November), 227 cases; winter group (January, February and December), 366 cases. The data was analyzed by using the Statistical Package for the Social Sciences for Windows (SPSS, version 11.0) to determine whether there was an association between age or season and the aetiological organism, and a p value <0.05 was considered to be statistically significant.

**Table 1:** **Socio-demographic profile of the study children**

**Age group**	**Age**	**Place of schooling**	**House condition**
Infants group	newborn ~1 year old	at home	spacious
toddlers group	>1 ~ 3 years old	at home	spacious
preschooler group	>3 ~ 6 years old	In daycare	crowded
school children	7 ~ 14 years old	In boarding school	crowded

### Blood sampling

Four milliliters of venous blood was drawn from each child. The samples were centrifuged at 2000 g for 10 min at 4°C. Serum was separated and stored at −20°C until assayed with the Pneumoslide IgM test.

### Pneumoslide IgM test (Vircell-slide, Granada, Spain)

Each slide has 10 wells with each well containing one of the following antigens: *Legionella pneumophila* serogroup 1 (LP1), *Mycoplasma pneumoniae* (MP), *Coxiella burnetii* (COX), *Chlamydophila pneumoniae* (CP), Adenovirus (ADV), Respiratory syncytial virus (RSV), Influenza A (INFA), Influenza B (INFB), Parainfluenza 1, 2 and 3 (PIVs) and a cell control. Serum samples were diluted 1:1 with phosphate buffered saline (PBS), and then treated with anti-human IgG sorbent. The sorbent-treated diluted serum was incubated 90 min at 37°C with the 10 slide wells. The slide was washed twice with PBS before the fluorescent secondary IgM antibody was added to the wells and incubated at 37°C for 30 minutes. The slide was washed twice with PBS and the greenish-yellow fluorescent signal was detected with a fluorescence microscope (Zeiss, Oberkochen, Germany).

## Results

### The positive percentage of 9 pathogens

Of a total of 1204 samples tested, 624 samples were positive for a positive percentage of 51.83%. The most predominant pathogen was *M. pneumoniae*, with a positive percentage of 40.78%. The pathogens ranking second to fourth place were INFB, PIVs, and RSV, with positive percentages of 7.06%, 4.82%, and 3.32%, respectively. Among all of the samples, only 1 COX and 1 INFA infection were detected (Table 2).

**Table 2:** **The positive percentages of 9 pathogens**

**Pathogens**	**Number**	**Positive percentage (%)**
*Mycoplasma pneumonia* (MP)	491	40.78
Influenza B (INFB)	85	7.06
Parainfluenza 1, 2 and 3 (PIVs)	58	4.82
Respiratory syncytial virus (RSV)	40	3.32
Adenovirus (ADV)	13	1.08
*Legionella pneumophilasero group* 1 (LP1)	11	0.91
*Chamydophila pneumonia* (CP)	4	0.33
*Coxiellaburnetii* (COX)	1	0.08
Influenza A (INFA)	1	0.08

### The positive percentages of pathogens isolated from different age groups

As a whole, the positive percentages of the 9 pathogens were 24.45% in infants, 47.38% in toddlers, 79.40% in preschoolers and 80.71% in school aged children. The positive percentages of viral and atypical bacterial pathogens increased with the rising ages of children. For the 4 major pathogens, the positive percentages of MP, INFB, PIVs and RSV were found to be associated with age, with p values of <0.001, <0.001, <0.001, and 0.038, respectively. *M. pneumoniae* was the most predominant pathogen in all age groups, compared to other pathogens. The positive percentages of MP and INFB in preschooler group were 55.33% and 12.41%, which were the highest, compared to other age groups, but the positive percentages of these two pathogens were just 13.04% and 2.17% in infants group. The positive percentage of PIVs in school children group was higher than that in other age groups and it was seen that the positive percentages increased with the age of patients (0.54% in infants group; 1.68% in toddlers group; 8.19% in preschool group; 11.43% in school group) (Table 3).

**Table 3:** **The positive percentages of pathogens isolated from different age groups with PneumoslideIgM test**

**Pathogens**	**Infants (%) N = 184**	**Toddlers (%) N = 477**	**Preschooler (%) N = 403**	**School children (%) N = 140**	***X*** ^**2**^	**P**
MP	24 (13.04)	169 (35.43)	223 (55.33)	75 (53.57)	109.105	<0.001
INFB	4 (2.17)	19 (3.98)	50 (12.41)	12 (8.57)	31.624	<0.001
PIVs	1 (0.54)	8 (1.68)	33 (8.19)	16 (11.43)	40.923	<0.001
RSV	11 (5.98)	19 (3.98)	7 (1.74)	3 (2.14)	8.450	0.038
ADV	4 (2.17)	5 (1.05)	3 (0.74)	1 (0.71)		
LP1	0 (0.00)	6 (1.26)	4 (0.99)	1 (0.71)		
CP	0 (0.00)	0 (0.00)	0 (0.00)	4 (2.86)		
COX	1 (0.54)	0 (0.00)	0 (0.00)	0 (0.00)		
INFA	0 (0.00)	0 (0.00)	0 (0.00)	1 (0.71)		

MP: Mycoplasma pneumonia; INFB: Influenza B; PIVs: Parainfluenza 1, 2 and 3; RSV: Respiratory syncytial virus; ADV: Adenovirus; LP1: Legionella pneumophilaserogroup 1; CP: Chamydophila pneumonia; COX: Coxiellaburnetii; INFA: Influenza A.

### The positive percentages of pathogens isolated from different seasons

**Figure 1:**
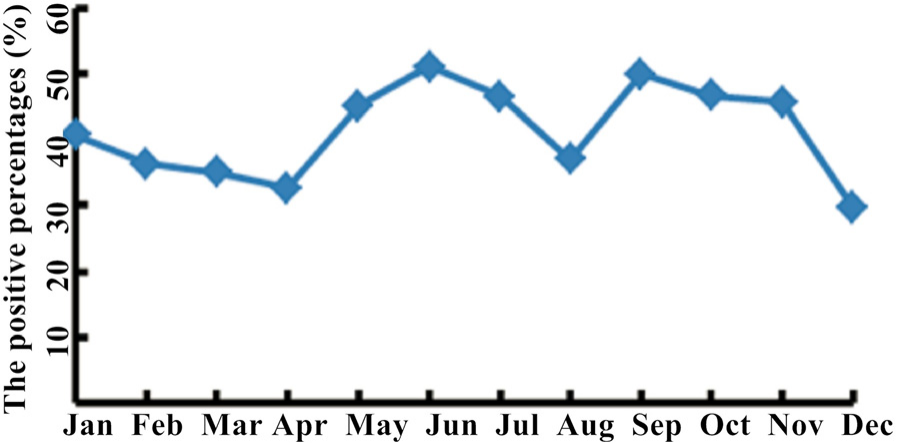
**The positive percentages of MP from January to December.** The positive percentages of MP were always high through the seasons (>29.63%).

**Figure 2:**
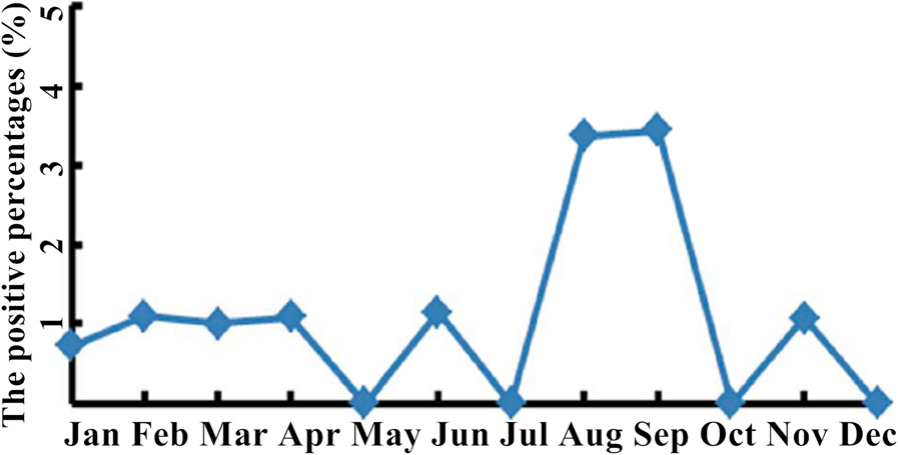
**The positive percentages of LP1 from January to December.** The positive percentages of LP1 in August and September were higher than in other months.

**Figure 3:**
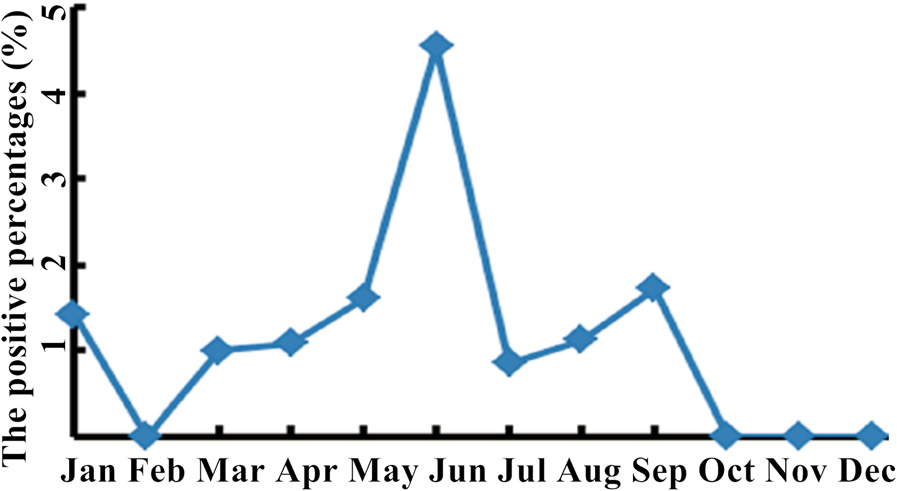
**The positive percentages of ADV from January to December.** The positive percentages of ADV in June were higher than in other months.

Among the 4 major respiratory tract pathogens, the positive percentages of MP, PIVs and RSV were found to be associated with seasons (MP, p = 0.011; PIVs, p = 0.009; RSV, p = 0.038) but the positive percentage of INFB was not found to be associated with season (p = 0.063) (Table 4). The positive percentage of MP was always high throughout the seasons (>29.63%) (Figure 1), compared to the other 8 pathogens. The positive percentages of MP was relatively high in summer and autumn (45.08% and 47.14% respectively) and relatively low in spring and winter (38.29% and 35.52% respectively) (Table 4). The positive percentages of LP1 in August and September and of ADV in June were higher than in other months (Figures 2 and 3).

**Table 4:** **The positive percentages of pathogens isolated from different seasons with PneumoslideIgM test**

**Pathogens**	**Spring (%) N = 316**	**Summer (%) N = 295**	**Autumn (%) N = 227**	**Winter (%) N = 366**	***X*** ^**2**^	**P**
MP	121 (38.29)	133 (45.08)	107 (47.14)	130 (35.52)	11.067	0.011
INFB	30 (9.49)	14 (4.75)	20 (8.81)	21 (5.74)	7.296	0.063
PIVs	23 (7.28)	12 (4.07)	15 (6.61)	8 (2.19)	11.651	0.009
RSV	14 (4.43)	3 (1.02)	6 (2.64)	17 (4.64)	8.408	0.038
ADV	4 (1.27)	6 (2.03)	1 (0.44)	2 (0.55)		
LP1	2 (0.63)	4 (1.36)	3 (1.32)	2 (0.55)		
CP	0 (0.00)	1 (0.34)	3 (1.32)	0 (0.00)		
COX	0 (0.00)	0 (0.00)	1 (0.44)	0 (0.00)		
INFA	1 (0.32)	0 (0.00)	0 (0.00)	0 (0.00)		

MP: Mycoplasma pneumonia; INFB: Influenza B; PIVs: Parainfluenza 1, 2 and 3; RSV: Respiratory syncytial virus; ADV: Adenovirus; LP1: Legionella pneumophilaserogroup 1; CP: Chamydophila pneumonia; COX: Coxiellaburnetii; INFA: Influenza A.

Spring includes Mar, Apr, and May; summer includes Jun, Jul, and Aug; autumn includes Sep, Oct and Nov; winter includes Jan, Feb and Dec.

### The mixed infection among these respiratory tract pathogens

Of the 1204 samples tested, 79 were confirmed as mixed infection, for a 6.56% positive percentage. Generally, there were four major mixed infection types. There were 35 samples infected with MP and INFB (44.30% positive percentage) and 36.71% of these samples infected with MP and PIVs. Other mixed infection types were MP with RSV and INFB with PIVs. Sally, et al. found that MP often participated in mixed infections, and, according to one of their hypotheses, patients infected with MP may be susceptible to other infectious pathogens [10]. However, neither the present study nor previous findings could explain these results, so further research is needed to elucidate the question.

## Discussion

Some pathogens may have a higher seasonal incidence than others in certain geographic areas, and the diagnosis of those causing prevalent diseases would be of value. It was therefore important to know the aetiology of pneumonia in Nanjing, and to determine whether there was an association between age or season and aetiological organism. Among children in Nanjing, there were 4 major viral and atypical bacterial pathogens causing CAP that were detected with the Pneumoslide IgM test. *M. pneumoniae* was the most predominant pathogen in different age and seasonal groups, which did not agree with previous results. Ren, et al. found that INF was the most predominant pathogen among the Beijing population [11], although this difference may result from the different geographic area and different population. MP is highly contagious and can spread between people through bodily fluids and airborne droplets from sneezing and coughing. It was most easily spread among people who were in close contact with one another [12]. In China, children go to daycare when they are about 3 years old and go to primary school when they are about 7 years old. Compared to the spacious house conditions for infants and toddlers, the preschool and school-aged children lived or studied in densely populated (crowded) spaces because of the large population (Table 1) and MP was most easily spread among children. Our results showed a significant association among age groups (p < 0.01) (Table 3). The positive percentages of MP in the preschool and school groups were higher than the infants or toddlers groups. Although MP infections can occur at any time of year, they were most common in the late summer and autumn. Our results confirmed the epidemiological characteristics of MP; the positive percentages of MP in summer and autumn were 45.08% and 47.14%, respectively, and MP was significantly associated with seasons (p < 0.01) (Table 4). Patients with MP infection may be susceptible to infection with other pathogens, resulting in mixed infections, such as MP with INFB or MP with PIVs. When treating a mixed infection, particular attention must be paid to the characteristics of the infection and the responsible pathogens.

Influenza is an RNA virus of the *orthomyxoviridae* family and has three serotypes (A, B, and C) which have been described [13,14]. In the samples tested, only 1 INFA positive was detected. However 85 samples infected with INFB were detected in these same subjects (Table 2). INFB usually causes disease in populations confined to closed spaces, such as daycare centers and boarding schools [15]. Our results show the positive percentages of INFB in the preschool and school children groups were higher than that in the infants and toddlers groups (p < 0.01) (Table 3).

PIVs can spread from person to person through close contact [16,17]. Like MP and INFB, children who lived in crowed space (such as daycare and boarding school conditions) were susceptible to infect with PIVs (p < 0.01) (Table 3). PIVs infections are most common in the spring, summer and fall, and our results were concordant with the characteristics of PIVs (p < 0.01) (Table 4).

RSV was the most common viral cause of lower respiratory infections in children, especially children younger than 2 years, and was rarely diagnosed in adults [8,18,19]. Our results show a higher association for RSV infection among the infants and toddlers groups (p < 0.05), although the positive percentages of RSV in the infants and toddlers groups were only 5.98% and 3.98, respectively (Table 3). RSV was found to be associated with season (p < 0.05) and the positive percentages of RSV were higher in the spring and winter seasons (Table 4).

## Conclusions

As observed in our results, there were 4 major viral and atypical bacterial pathogens causing CAP, and some pathogens were found to be associated with age and season. *M. pneumoniae* was the dominant pathogen. Crowed spaces (daycare, boarding school, etc.) may be the major reason for the spread of MP, INFB, and PIVs among children in Nanjing. The study accumulated valuable data about the aetiology and epidemiology of CAP among children in Nanjing, not only increasing the knowledge base of the aetiology of CAP, but also assisting in better planning of therapeutic and prevention strategies to prevent the spread of the pathogens in susceptible age groups during peak season.

### Limitation

The Pneumoslide IgM test has reasonable sensitivity and specificity for the detection of pathogens. However, the test could not detect bacterial pathogens and, therefore, the aetiology presented by the study were only for viral and atypical bacterial pathogens causing community-acquired pneumonia. In order to improve the positive percentages of detection for CP and COX, multiple serum samples with different sampling times can be tested to account for the later increase in antibody levels, compared to other pathogens.
